# Response to: Clinical Conundrum- an insignificant impact of statins on overall plaque volume regression with promotion of calcification?

**DOI:** 10.1161/CIRCIMAGING.121.012514

**Published:** 2021-04-20

**Authors:** Mohammed N. Meah, Mhairi K. Dorris, David E. Newby, Philip Adamson

**Affiliations:** 1 British Heart Foundation Centre for Cardiovascular Science, University of Edinburgh, Edinburgh, UK; 2 Edinburgh Imaging, Queen’s Medical Research Institute University of Edinburgh, Edinburgh, UK; 3 Christchurch Heart Institute, University of Otago, Christchurch, NZ

We would like to thank Drs Bass and Garcia-Gracia for their interest in our work and for their complimentary remarks. They raise several important points relating to the interpretation of 18F-sodium fluoride uptake and progression of calcification in relation to the use of statin therapy and the presence of inflammation.

From our study, it is impossible to distinguish the effects of statins given that our participants had advanced disease, 95% were on intensive statin therapy and there was no control group. The absence of an effect on non-calcific plaque burden is likely to reflect the chronicity of statin therapy as well as proof of efficacy given the absence of an increase in non-calcific plaque volume. Our findings are therefore consistent with the previous studies cited by Drs Bass and Garcia-Gracia where plaque volumes were compared with statin-naïve patients who had progression of disease. It is also important to appreciate that the intravascular ultrasound studies have a number of confounders including the use of non-randomized data and survival bias.^1^

The main question to address is whether coronary calcification is good or bad: we believe it is both! Coronary calcification needs to be considered not just in terms of absolute score but also by its pattern of distribution and density. Motoyama and colleagues reported that spotty calcifications are closely associated with plaque rupture events and others reported that lower calcium density is also associated with higher cardiovascular risk.^2,3^ In contrast, marked individual plaque calcification is associated with more stable disease.^2^ Intuitively, extensive macrocalcification of the coronary plaque will stabilise the plaque surface, constrain the lipid-rich necrotic core and prevent plaque rupture. Indeed, this probably explains why statins both stabilise plaque and increase coronary calcification: something we reported 15 years ago and confirmed in recent meta-anlyses.^4^ So how do we square the fact that the presence of, and increase in, coronary calcification predicts risk but the calcification itself is associated with plaque stabilisation? Of course, the extent of calcification reflects disease burden, and this underlies the association with future events.^5^ No or little calcification indicates the absence or minimal presence of coronary atheroma. Progression of atherosclerosis will cause more calcification although there will clearly be a time delay: this is a chronic process stimulated by necrotic plaque and inflammation. 18F-Sodium fluoride identifies active (micro)calcification that leads to progression of macrocalcification: a measure of both the presence of lipid-rich necrotic plaque and the healing response to it. If it occurs in the early phase of healing, it will identify a highly unstable plaque. In the latter stages of disease, it may identify a plaque completing its calcification process. Ultimately, 18F-sodium fluoride is a marker of the response to an inflammatory stimulus rather than a mediator of the inflammation itself.
